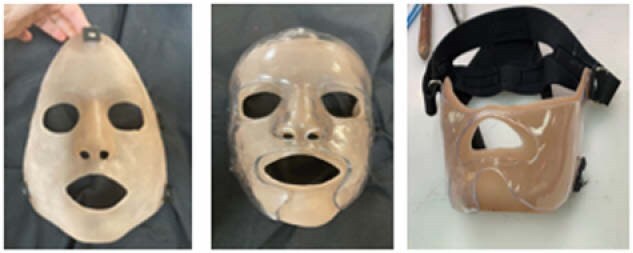# 992 Optimizing Facial Outcomes: A One-Year Evaluation of the Sequential Compression Appliance to Reduce Scarring Facemask

**DOI:** 10.1093/jbcr/iraf019.523

**Published:** 2025-04-01

**Authors:** Michelle Dwertman, Dr Henry Huson

**Affiliations:** University of Cincinnati Medical Center Burn Center; University of Cincinnati Burn Center

## Abstract

**Introduction:**

The management of facial scarring remains a critical challenge with the limitations of traditional transparent facial orthoses (TFOs), which are rigid, lack the ability to adapt to facial movements, and are difficult to customize. To overcome these obstacles, we developed a ‘Sequential Compression Appliance to Reduce Scarring’ (SCARS) facemask - a novel orthosis designed to provide effective scar management, while allowing natural facial movement. This study builds on our previous work by further evaluating the SCARS mask’s design, and clinical outcomes.

**Methods:**

Patients were fitted with a traditional TFO outer layer and a SCARS flexible inner layer made of Heat Cure Rubber (HCR) silicone. The inner SCARS layer delivers a minimum pressure of 20mmHg, conforming to facial contours, while maintaining flexibility for movements with the temporomandibular joint (TMJ). Customizations include color tinting to match individual skin tones and a lightweight design that allows the SCARS mask to be worn independently or in conjunction with the TFO. Further, it may be worn with a rigid neck collar, has widely available harnessing, and trimming may be done with scissors. The SCARS mask is molded and shaped based on a 3D scan. Trials were conducted on three patients with severe facial burns and microstomia. Trials assessed patient scarring, preference, comfort during wear, and range of motion (ROM) while using the SCARS mask. Two patients were unable to tolerate the traditional rigid TFO and solely utilized the SCARS inner layer.

**Results:**

Patients using the SCARS masks showed a 25% reduction in scar height and a 12% improvement in skin pliability, as measured by the Vancouver Scar Scale. Patients reported a strong preference for the SCARS mask over traditional TFOs, citing improved comfort and freedom of movement, with an average Likert scale rating of 4.5. The SCARS mask’s ability to accommodate natural facial movements was especially beneficial during eating, where the TFO made eating impossible (average rating: 5), but the SCARS mask allowed for mild difficulty (average rating: 2.43).

**Conclusions:**

The SCARS facemask offers a transformative approach to facial pressure management that is safe and effective for decreasing scar height and pliability, combining flexibility, adaptability, and patient-centered design. Its innovative features not only enhance patient comfort and access, but also provide effective scar treatment, making it a valuable option in clinical practice for individuals with facial burn-related scarring.

**Applicability of Research to Practice:**

The SCARS mask’s pliable design offers a viable option for managing facial scarring, with potential to improve patient adherence. Its compatibility with rigid neck collars, use with widely available harnessing, color-tinting, and cost-effective modifications make it an accessible and practical solution for enhancing scar management outcomes.

**Funding for the Study:**